# The role of microRNA-142a in *Toxoplasma gondii* infection-induced downregulation of Foxp3: implications for adverse pregnancy outcomes

**DOI:** 10.1186/s12879-024-09375-0

**Published:** 2024-05-13

**Authors:** Yue Zhong, Yining Cao, Xiaoyu Geng, Shujin Yang, Tianmei Qian, Chun Liu, Jinling Chen

**Affiliations:** 1 Department of Pathogen Biology, School of Medicine, Nantong University, 19 Qixiu Road, Nantong, Jiangsu, 226001 People’s Republic of China; 2 Laboratory Animal Center, Nantong University, 19 Qixiu Road, Nantong, Jiangsu, 226001 People’s Republic of China; 3 ZhenJiang Provincial Blood Center, Zhenjiang, Jiangsu, 212000 People’s Republic of China; 4 Engineering Research Center of Integration and Application of Digital Learning Technology, Ministry of Education, Beijing, 100034 People’s Republic of China; 5 NMPA Key Laboratory for Research and Evaluation of Tissue Engineering Technology Products, Nantong University, Nantong, Jiangsu, 226001 People’s Republic of China

**Keywords:** *Toxoplasma Gondii*, microRNA-142a-3p, Forkhead box P3, P53

## Abstract

**Background:**

*Toxoplasma gondii* (*T. gondii*) is capable of infecting nearly all warm-blooded animals and approximately 30% of the global population. Though most infections are subclinical in immunocompetent individuals, congenital contraction can lead to severe consequences such as spontaneous abortion, stillbirth, and a range of cranio-cerebral and/or ocular abnormalities. Previous studies reported that *T. gondii*-infected pregnancy mice unveiled a deficit in both the amount and suppressive functions of regulatory T (Treg) cells, accompanied with reduced levels of forkhead box p3 (Foxp3). Recently, accumulative studies have demonstrated that microRNAs (miRNAs) are, to some extent, relevant to *T. gondii* infection. However, the link between alterations in miRNAs and downregulation of Foxp3 triggered by *T. gondii* has been only sporadically studied.

**Methods:**

Quantitative reverse transcription polymerase chain reaction (RT-qPCR), protein blotting and immunofluorescence were employed to evaluate the impact of *T. gondii* infection and antigens on miRNA transcription and Foxp3 expression. Dual-luciferase reporter gene assays were performed to examine the fluorescence activity in EL4 cells, which were transfected with recombinant plasmids containing full-length/truncated/mutant microRNA-142a-3p (miR-142a) promoter sequence or wild type/mutant of Foxp3 3’ untranslated region (3’ UTR).

**Results:**

We found a pronounced increase in miR-142a transcription, concurrent with a decrease in Foxp3 expression in *T. gondii*-infected mouse placental tissue. Similarly, comparable findings have been experimentally confirmed through the treatment of EL4 cells with *T. gondii* antigens (*Tg*Ag) in vitro. Simultaneously, miR-142a mimics attenuated Foxp3 expression, whereas its inhibitors markedly augmented Foxp3 expression. miR-142a promoter activity was elevated upon the stimulation of *T. gondii* antigens, which mitigated co-transfection of mutant miR-142a promoter lacking P53 target sites. miR-142a mimics deceased the fluorescence activity of Foxp3 3’ untranslated region (3’ UTR), but it did not affect the fluorescence activity upon the co-transfection of mutant Foxp3 3’ UTR lacking miR-142a target site.

**Conclusion:**

In both in vivo and in vitro studies, a negative correlation was discovered between Foxp3 expression and miR-142a transcription. *Tg*Ag enhanced miR-142a promoter activity to facilitate miR-142a transcription through a P53-dependent mechanism. Furthermore, miR-142a directly targeted Foxp3 3’ UTR, resulting in the downregulation of Foxp3 expression. Therefore, harnessing miR-142a may be a possible therapeutic approach for adverse pregnancy caused by immune imbalances, particularly those induced by *T. gondii* infection.

## Background

*Toxoplasma gondii* (*T. gondii*), belonging to a cosmopolitan protozoon, can infect a plethora of warm-blooded animals including humans and felines [1, 2]. It is estimated that, globally, nearly 30% of the global population is exposed to *T. gondii* infection [3]. *T. gondii* has a relatively high rate of congenital transmission, which occurs mostly during the first trimester of pregnancy with maternal infections, causing neonatal mortality or stillbirth, growth retardation, blindness, and encephalitis [4, 5]. Regulatory T (Treg) cell, as a component of the canonical CD4^+^ T cell, is an indispensable mediator for a sturdy placenta and sustainable pregnancy [6, 7]. The decreased percentages of CD4^+^CD25^+^ Treg cells were found in both peripheral blood and decidua of patients with unexplained recurrent spontaneous abortion (URSA), attesting to the importance of Treg cells in maintaining immunotolerance during normal pregnancy [8, 9]. The adoptive transfer of Treg cells from pregnant mice rather than non-pregnant mice remarkably improved pregnancy outcomes, further emphasizing the importance of Treg cells in ensuring successful pregnancies [10].

The transcription factor Forkhead box P3 (Foxp3) encodes a forkheaded-winged-helix transcription factor called Scurfin, which is considered to be essential for mounting immunosuppressive functions in Treg cells [11, 12]. Foxp3 is highly expressed in CD4^+^CD25^+^ Treg cells, aiding in the distinction between Treg cells and activated, non-regulatory T cells [13]. In a study of 150 women aged 38 years and younger with low levels of midluteal Foxp3^+^Tregs, endometrial Foxp3^+^Tregs levels were found to be profoundly higher after intrauterine injection of human chorionic gonadotropin (hCG), and the clinical pregnancy rate was noticeably higher than that of the control group (54.8% versus 74.0%) [14]. A decreased Foxp3^+^ Tregs infiltrating in the midluteal phase was closely related to human unexplained infertility [15]. The importance of Foxp3^+^Tregs is further supported by a preclinical study demonstrating that fetal antigenic stimulation drives maternal Foxp3^+^Tregs expansion, whereas Foxp3^+^Treg depletion leads to fetal death [16]. Single nucleotide polymorphisms (SNPs) within Foxp3 promoter region, might influence Foxp3 protein expression, where both − 3279 C/A (rs3761548) and − 924 A/G (rs2232365) are linked to the risk of immune-associated pregnancy complications. Women carrying G allele have a higher risk of URSA than those carrying the A allele [17]. Thus, aberrant transcription of Foxp3 may be a significant contributing factor in immune-related pregnancy complications.

microRNAs (miRNAs), belonging to a class of endogenous, non-coding RNAs, act on post-transcription regulation of gene expression, leading to messenger RNA (mRNA) degradation or inhibiting mRNA translation [18, 19]. Accumulating data in recent years have revealed that miRNAs are involved in *T. gondii* pathogenesis [20]. Human macrophages are subject to apoptosis due to the suppression of miR-20a in the case of *T. gondii* infection [21]. miR-155-5p, up-expressed in patients with ocular toxoplasmosis compared with asymptomatic individuals, can induce macrophage differentiation into diverse phenotypes when confronted with infection and inflammatory response [22]. Simultaneously, recent research supports the notion that miRNAs negatively modulate Foxp3 expression [23]. miR-182 knockdown in CD4^+^ T cells, which are isolated from draining lymph nodes of female mice, markedly enhanced Foxp3 mRNA expression and the percentages of Foxp3^+^ Treg cells in vitro [24]. A negative link between miR-125b and Foxp3 expression was also confirmed in tissue samples and cell lines of thyroid cancer. miR-125b negatively governs Foxp3 expression via direct binding to Foxp3 3’ UTR, promoting autophagy and enhancing the efficacy of cisplatin in thyroid cancer [25]. Our previous studies have demonstrated that *T. gondii* or antigens can cause adverse pregnancy outcomes in mice, which is partially owing to the insufficiency of Foxp3^+^ Treg cells [26]. Though previous studies have confirmed that miRNAs are key regulators of Foxp3 expression, the interaction of miRNAs with *T. gondii*-induced Foxp3 downregulation remains unclear.

In this study, leveraging an in vivo pregnant mouse miscarriage model, we found that the adverse pregnancy outcomes triggered by *T. gondii* infection are relevant to the alteration of miR-142a and the downregulation of Foxp3. Notably, *Tg*Ag negatively governs Foxp3 expression mainly through two different mechanisms: Firstly, *Tg*Ag promotes the transcriptional activity of miR-142a promoter in a P53-dependent manner. Secondly, miR-142a represses Foxp3 expression via direct binding to its 3’UTR. Collectively, harnessing miR-142a might be a potential therapeutic approach for adverse pregnancy outcomes caused by immune imbalances, especially those resulting from *T. gondii* infection.

## Materials and methods

### Ethics approval

Mouse experiments in the current study were performed according to the Regulations of the People’s Republic of China on the Administration of Laboratory Animals (licence number: 20210304-010). Mice were housed under standard conditions, ensuring their welfare and well-being. Mice were euthanized via CO_2_ asphyxiation [27].

### *T. gondii* antigen preparation

*T. gondii* tachyzoites (RH strain) were maintained in ICR mice via intraperitoneal passage at 72 h intervals. *Tg*Ag was prepared according to the report by Qiu et al. [28]. Tachyzoites (1 × 10^8^) were then incubated in 10 mL of serum-free RPMI 1640 medium (Thermo Fisher Scientific, Waltham, MA, USA) for 3 h at 37 $^ \circ {\rm{C}}$ with gentle agitation. *Tg*Ag-containing supernatant was harvested, centrifuged at 1000 × g for 10 min at 4 $^ \circ {\rm{C}}$, and then concentrated by using an Amicon Ultra-15 centrifugal filter unit (Merck Millipore, Darmstadt, Germany). AffinityPak Detoxi-Gel endotoxin removal kit (Thermo Fisher Scientific) was applied to remove the endotoxin in *Tg*A*g*. The concentration of endotoxin in *Tg*Ag was < 0.1 EU/mg protein as determined by Limulus assay (Xiamen Limulus Reagent Co., Ltd., Fujian, China). The *Tg*Ag preparation underwent sterilization using a 0.22 μm filter (Merck Millipore), and the final concentration was determined by the Bradford method.

### Mouse model

ICR mice (six to eight-week-old), purchased from the Animal Research Center of Nantong University, were provided with diets and water *ad libitum*. Female and male mice were paired in a 2:1 ratio. At 7 am the next morning, the presence of a vaginal plug in female mice was considered as embryonic day 0.5 (E0.5) [29]. At E8.5, approximately 500 tachyzoites were intraperitoneally injected into the pregnant mice. Then, all mice were euthanized and sacrificed at E18.5.

### Cell treatment and reagents

EL4 cell, a murine T lymphoma cell, was ordered from Cell Bank of Shanghai Institute and cultured in completed DMEM (Thermo Fisher Scientific) supplemented with 10% fetal bovine serum (FBS, Excellbio, Shanghai, China), penicillin (100 IU/mL), and streptomycin (100 µg/mL). EL4 cells were cultured in a humidified incubator with 5% CO_2_ at 37 °C and passaged when the fusion reached 70%. EL4 cells were first stimulated with anti-CD3 (precoated), TGF-β (5 ng/mL), and anti-CD28 (1 µg/mL), followed by the *Tg*Ag (10 µg/mL) treatment for 24 h.

### RNA isolation and RT-qPCR

Small RNA, isolated from EL4 cells or placenta tissues with RNAiso (Takara, Kyoto, Japan), was reverse transcribed to generate cDNA by using Mir-X™ miRNA First-Strand Synthesis Kit (Takara). RT-qPCR was conducted on a StepOne™ real-time PCR system (Applied Biosystems, Foster City, CA, USA) using SYBR Green Premix Ex Taq™ II (Promega, Madison, WI, USA). mmu-miR-142a-3p primer was 5′-CGTGTTCACAGCGGACCTTGAT-3′. The U6 small nuclear RNA served as endogenous control for miRNAs and Ct value of the relative genes was assayed by using the 2^−△△Ct^ method.

### Western blot

Cells were harvested and lysed in ice-cold lysis buffer containing protease inhibitors. Proteins were separated via 8-10% sodium dodecyl sulfate-polyacrylamide gel electrophoresis. Proteins on the gel were then transferred to polyvinylidene fluoride (PVDF) membranes (Merck Millipore), followed by the blockage with 5% skimmed milk for 1 h at 20 $^ \circ {\rm{C}}$ and incubation overnight at 4 $^ \circ {\rm{C}}$ with primary antibodies diluted in TBST. Primary antibodies were: Foxp3 (Abcam, Cambridge, UK, 1:1000), P53 (CST, Danvers, MA, USA, 1:1000), GR (Proteintech, Rosemont, USA, 1:2000), Foxo3 (Proteintech, 1:2000) and GAPDH (Proteintech, 1:10000). After three washes with TBST, PVDF membrane was incubated with horseradish peroxidase (HRP)-conjugated secondary antibodies for 2 h at 20 $^ \circ {\rm{C}}$. Antigen-antibody immune complex was visualized by enhanced chemiluminescence (ECL, Meilunbio, Dalian, China). Greyscale analysis was performed using Image J software in Western blot.

### miRNA transient transfection

All miRNA inhibitors and mimics were synthesized by Gene Pharma (Shanghai, China). “mmu” represents mus musculus, and “NC” represents the negative control. NC mimic: F: 5’-UUCUCCGAACGUGUCACGUTT-3’; R: 5’- ACGUGACACGUUCGGAGAATT-3’; NC inhibitor: F: 5’-CAGUACUUUUGUGUAGUACAA-3’; mmu-miR-142a mimic: F: 5’-UGUAGUGUUUCCUACUUUAUGGA3’; R: 5’-CAUAAAGUAGGAAACACUACAUU; mmu-miR-142a inhibitor: 5’-UCCAUAAAGUAGGAAACACUACA-3’. For transfection assay, EL4 cells were first incubated in 6-well plates containing completed DMEM medium, followed by the treatments with miR-142a mimic (20 µM), miR-142a inhibitor (20 µM) as well as their negative controls using electroporation system (BTX Harvard Apparatus, Holliston, MA, USA) in line with the manufacturer’s instructions, respectively. After 48 h of incubation, cells were harvested for further experiments.

### Immunofluorescence staining

For immunofluorescence staining (IF) analyses, samples were fixed in 4% paraformaldehyde for 30 min. After three washes with PBS, samples were incubated in blocking buffer containing BSA (5%) and Triton X-100 (0.5%). For P53 staining, samples were incubated with anti-P53 antibody (1:100, CST) in PBS at 4 $^ \circ {\rm{C}}$ overnight. Alexa Fluor 488-conjugated anti-mouse IgG (Thermo Fisher Scientific) was incubated with the samples for 2 h at 20 $^ \circ {\rm{C}}$, while avoiding exposure to light. The slides, labeled with 4’,6-diamidino-2-phenylindole (DAPI) to highlight the nucleus, were observed under a Leica microscope, and images were captured using software.

### Plasmid constructs

The binding sites between Foxp3 3’UTR and miR-142a were predicted by bioinformatics sites including miRWalk (http://mirwalk.umm.uni-heidelberg.de/) and TargetScan (http://www.targetscan.org/vert_72/). To confirm the direct inhibition of Foxp3 by miR-142a, the primers of Foxp3 3’ UTR (GenBank accession no. NM_ 001199347, http://mirwalk.umm.uni-heidelberg.de/mouse/gene/20371/) containing restriction sites of *Xho* I and *Not* I, which were listed in Table 1, were designed by Primer 5 software. The mutant of Foxp3 3’ UTR was generated via an overlap PCR using the primers listed in Table 1. The sequence of Foxp3 3’ UTR comprising wild-type (WT) or mutant (MUT), amplified by PCR, was subcloned into psiCHECK-2 vector to construct recombinant plasmids.

**Table 1:** Primers required for constructing recombinant plasmids containing Foxp3 3’UTR fragments or miR-142a promoter sequences

Plasmid	Restriction enzyme	Primer sequence
WT-Foxp3 3’-UTR	*Xho*I *Not*I	Forward: CCGCTCGAGTCAAAGTCTTCTTGCCCATCTCTGT Reverse: ATAAGAATGCGGCCGCAAGCCTTGTATTCTTGCTGTCTCCA
MUT-Foxp3 3’-UTR	*Xho*I *Not*I	Forward: TTCTGTTTTTTTTTTTTTTTTTTTTTTTTTGCCGC Reverse: AAGACAAAAAAAAAAAAAAAAAAAAAAAAATGGCG
pGL3-E-142a	*Kpn*I *Hind*III	Forward: CGGGGTACCATGAAGACCCAATCCAGTTGTGAC Reverse: CCCAAGCTTATGCTCACCTGTTTCCTTGATGTT
pGL3-E-142a-A	*Kpn*I *Hind*III	Forward: CGGGGTACCGCTTTGCTTGGTGGCAGTGA Reverse: CCCAAGCTTATGCTCACCTGTTTCCTTGATGTT
pGL3-E-142a-B	*Kpn*I *Hind*III	Forward: CGGGGTACCATAAGCCCTACCCACCCTGAAG Reverse: CCCAAGCTTATGCTCACCTGTTTCCTTGATGTT
pGL3-E-142a-C	*Kpn*I *Hind*III	Forward: CGGGGTACCGGGAGGCAGAGACAGATGGATT Reverse: CCCAAGCTTATGCTCACCTGTTTCCTTGATGTT
pGL3-E-142a-A-Mut 1 F/R	*Kpn*I *Hind*III	Forward: GGCTGGAGCCATTGGGGAATTTTTTTCCTGGGAGCCAGGAAGCC Reverse: CCGACCTCGGTAACCCCTTAAAAAAAGGACCCTCGGTCCTTCGG
pGL3-E-142a-A-Mut 2 F/R	*Kpn*I *Hind*III	Forward: GCCAGGAAGCCACCTGCCCATTTTTTTTGAGATGGCCTCTTCAG Reverse: CGGTCCTTCGGTGGACGGGTAAAAAAAACTCTACCGGAGAAGTC

The primers of the full-length sequence and three truncated fragments of the miR-142a promoter (Table 1) were designed based on the miR-142a promoter (GeneBank no. NM_ 000083.7, https://www.ncbi.nlm.nih.gov/gene/387160) using Primer5 software. A 2.1 kb *kpn* I /*Hind* III fragment of -1950 to + 150 nucleotides corresponding to the identified miR-142a promoter transcription start site was subcloned into the pGL3-Enhancer vector, named pGL3-E-142a. The sequences comprising the truncated fragments in the miR-142a promoter region or the mutants of P53 binding sites in miR-142a promoter region, were subcloned into pGL3-Enhancer vector for the construction of recombinant plasmids, called pGL3-E-142a-A/B/C, pGL3-E-142a-A-Mut 1/Mut 2, and pGL3-E-142a-A-Mut 1 + 2. Transcription factor prediction databases, including PROMO (https://alggen.lsi.upc.es/cgi-bin/promo_v3/promo/promoinit.cgi?dirDB=TF_8.3/) as well as JASPAR (https://jaspar.genereg.net/*)*, were used to predict whether there are binding sites between miR-142a promoter region and transcription factors.

### Dual-luciferase sensor assay

EL4 cells were transiently co-transfected in 6-well plates with recombinant plasmids comprising WT or MUT of Foxp3 3’UTR together with miR-142a mimic/inhibitor as well as their negative controls. Recombinant plasmids comprising pGL3-E-142a, pGL3-E-142a-A/B/C, pGL3-E-142a-A-Mut 1/Mut 2, pGL3-E-142a-A-Mut 1 + 2, and the pRL-TK Renilla luciferase vector (Promega), were co-transfected into EL4 cells. *Tg*Ag treatment was performed at 24 h post-electroporation. After 48 h of transfection, luciferase activity of EL4 cells was measured by using luciferase assay kit (Promega). Relative luciferase activity of each group was normalized to the corresponding Firefly or Renilla luciferase activity.

### Statistical analysis

The data, pooled from at least three independent experiments, were shown as mean ± standard deviation (SD). All statistical analyses were performed with GraphPad Prism 8.0 software (GraphPad Inc, La Jolla, CA, USA). Two-group comparison was performed by using two-tailed unpaired Student’s *t*-test while multiple-group comparison was conducted by using one-way analysis of variance (ANOVA). Generally, statistical significance was assumed when probability (*P*) value ≤ 0.05.

## Result

### *T. gondii* and its antigens affect miR-142a transcription and Foxp3 expression in vivo and ex vivo

**Fig. 1:**
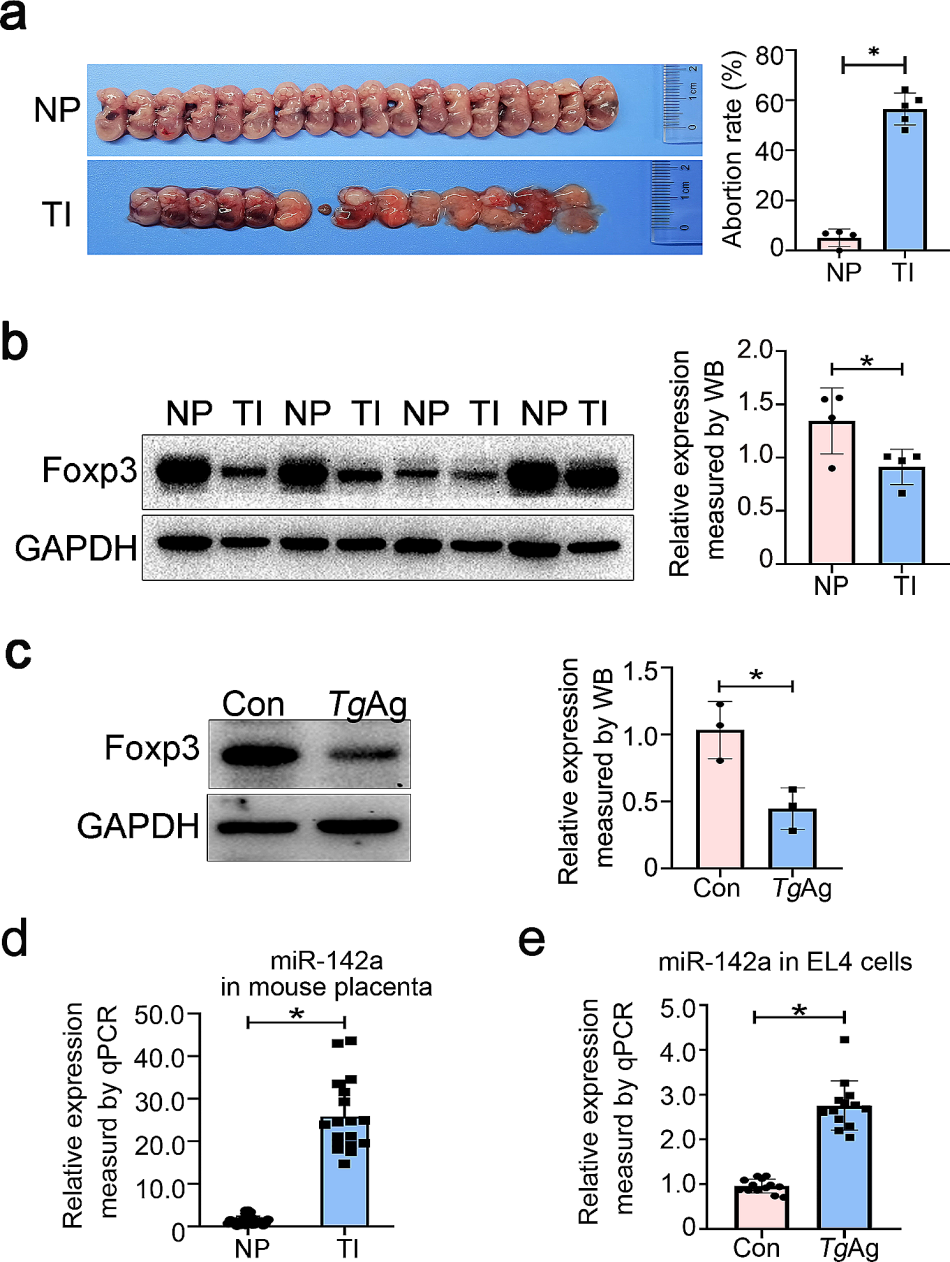
Effects of *T. gondii* infection on miR-142a transcription and Foxp3 expression In vivo and in vitro. **a** Diagram of mouse fetus at E18.5. In the normal pregnancy group (*n* = 4 mice), the embryonic mice developed normally, while in the *T. gondii* infection group (*n* = 5 mice), mouse fetuses appeared to be stillborn, with the abortion rate up to 60%. **b** Western blot analysis detected the changes in Foxp3 expression in mouse placental tissues. **c** Western blot was performed to assay the changes of Foxp3 protein expression in EL4 cells after 24 h of in vitro stimulation with *Tg*Ag. **d** RT-qPCR was used to assay the alterations of miR-142a expression in the placenta. **e** RT-qPCR was used to analyze the alterations of miR-142a expression in the EL4 cells stimulated with *Tg*Ag. NP: normal pregnant mice; TI: *Toxoplasma*-infected pregnant mice. *Tg*Ag: *T. gondii* antigens. Data were presented as mean ± SD. Statistical analysis was conducted using two-tailed unpaired Student’s *t*-test (B, C, D and E). *: *P* < 0.05

*T. gondii* can be transmitted directly to the fetus through the placenta resulting in neonatal miscarriages and fetal malformations [30]. In the present study, the pregnant mice were injected intraperitoneally at E8.5 with *T. gondii*. We divided the experiment into normal pregnancy group and *T. gondii*-infected group. As shown in Fig. 1a, the normal pregnant mice exhibited vigorous vigour, concurrent with normal development of the fetus and placenta. Adverse pregnancy outcomes were found in *T. gondii*-infected mice, exhibiting fetal mouse malformations and deaths. Collectively, these findings demonstrate that *T. gondii* infection results in adverse pregnancy outcomes in mice.

Foxp3 expression was declined in the placenta of *T. gondii*-infected mouse in comparison with normal placental tissue (Fig. 1b). Having demonstrated that *Tg*Ag played a pivotal role in the immune response to *Toxoplasma* [31], we tested, using Western blot, whether *Tg*Ag exerted robust inhibitory function on Foxp3 expression. We found that Foxp3 expression was markedly reduced in *Tg*Ag-stimulated EL4 cells in comparison with the control group (Fig. 1c).

Notably, some studies reported that *T. gondii* promoted significant expression of numerous miRNAs during the early stages of infection in mice [32]. In our study, *T. gondii* infection was found to cause the upregulation of miR-142a transcript in the mouse placenta, as determined by RT-qPCR (Fig. 1d). Under a 24-hour in vitro stimulation with *Tg*Ag, EL4 cells were collected. RNA was extracted and then reverse transcribed into cDNA. RT-qPCR was performed to assay the effect of *Tg*Ag on the transcription level of miR-142a gene. miR-142a transcript level was elevated in the *Tg*Ag-stimulated group (Fig. 1e). In conclusion, both *T. gondii* infection and antigens can inhibit Foxp3 protein level and promote miR-142a transcription in vitro and in vivo.

### miR-142a attenuates Foxp3 expression

**Fig. 2:**
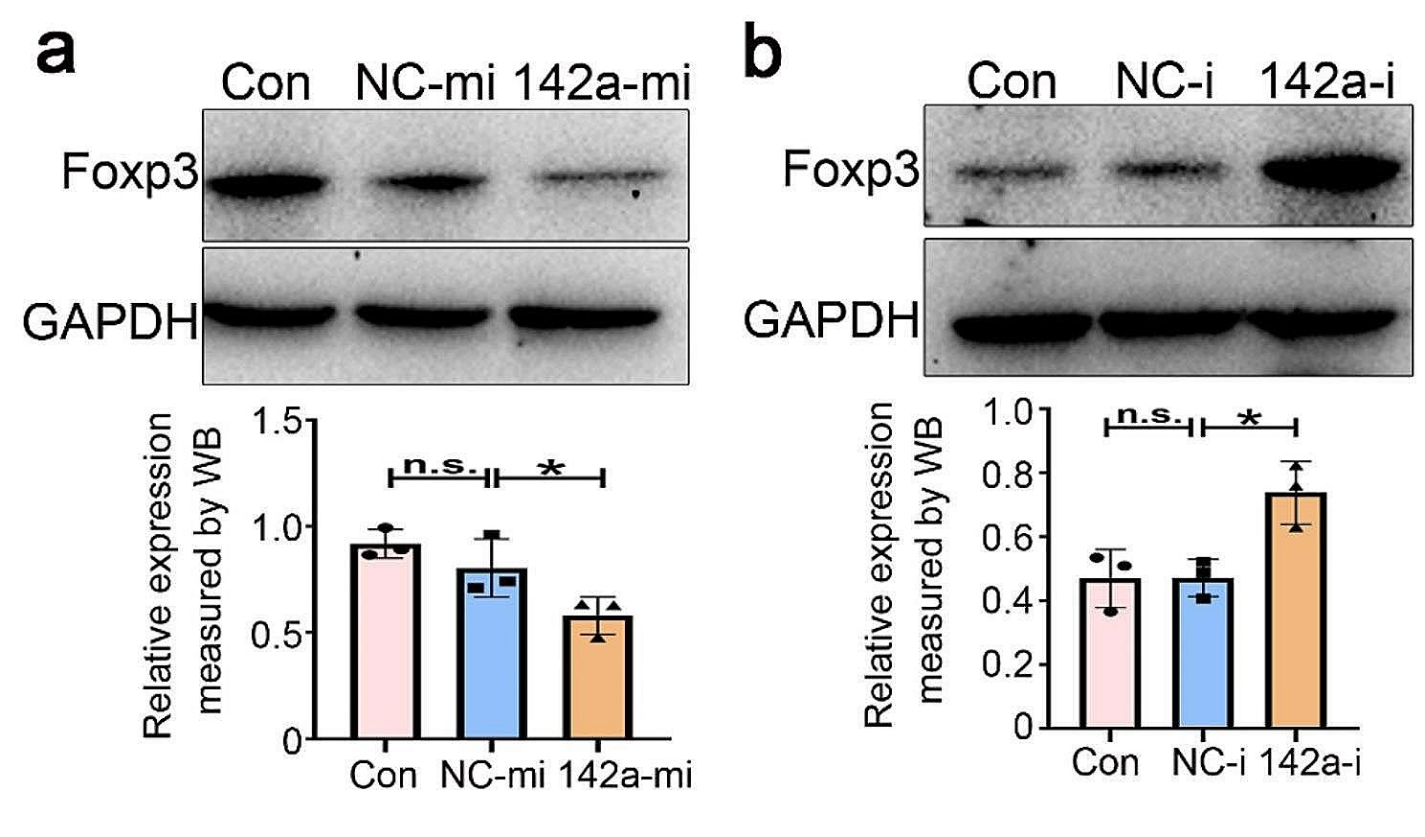
miR-142a attenuated Foxp3 expression. **a** miR-142a mimics were transfected into EL4 cells, and Foxp3 expressions were assayed by using immunoblotting. NC-mi: negative controls of miRNA mimics; miR-142a-mi: microRNA-142a mimics. **b** miR-142a inhibitors were transfected into EL4 cells, and Foxp3 expressions were detected by using immunoblotting. NC-i: negative controls of miRNA inhibitors. miR-142a-i: microRNA-142a inhibitors. Data were representative of the results of three independent experiments (mean ± SD). Statistical analysis was conducted using one-way ANOVA Tukey’s multiple comparisons test. *: *P* < 0.05; n.s.: *P* > 0.05

miRNAs can modulate the stability and translation of target genes at the post-transcriptional level [33]. Owing to further explore the effect of miR-142a on Foxp3 expression, miR-142a mimics, inhibitors, and their respective controls respectively transfected into EL4 cells. Western blot analysis indicated that Foxp3 expression was robustly declined in miR-142a mimic group in comparison with control group in Fig. 2a, whereas the miR-142a inhibitor group had the opposite effect on Foxp3 expression in Fig. 2b. Thus, this evidence suggests that miR-142a could repress Foxp3 expression.

### Antigens of *T. gondii* facilitate the promoter activity of miR-142a

**Fig. 3:**
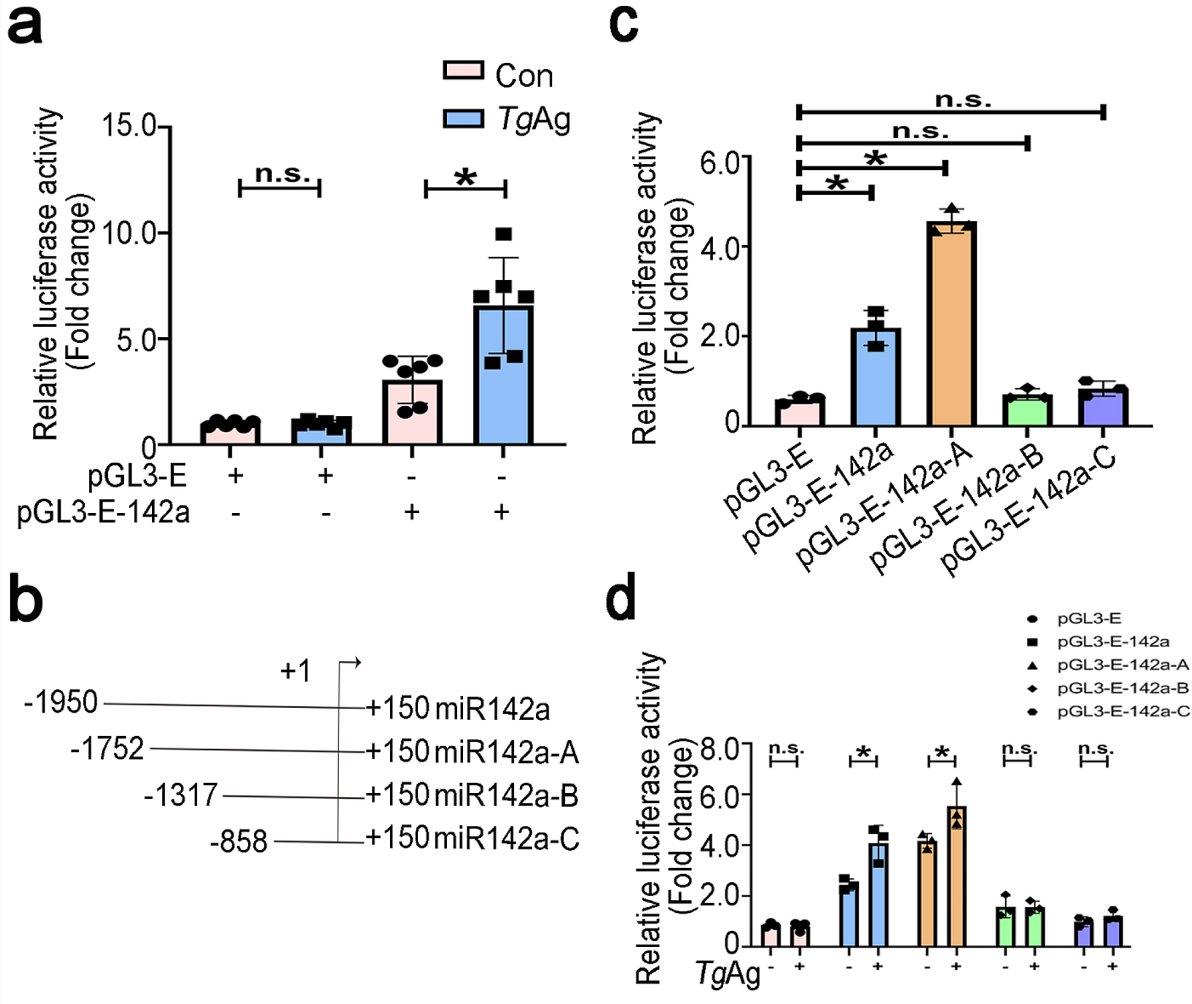
Antigens of *T. gondii* elevated the miR-142a promoter activity. **a** Luciferase reporter assay was conducted to measure the luciferase activity of EL4 cells that were electroporated with pGL3-E or pGL3-E-142a and then stimulated by *Tg*Ag for 24 h, respectively. **b** Schematic diagram showed the truncation sequences at the miR-142a promoter region. **c** The luciferase activities of EL4 cells, transfected respectively with recombinant plasmids comprising pGL3-E, pGL3-E-142a, and pGL3-E-142a-A/B/C, were evaluated at 48 h-post transfection. **d** The luciferase activities of EL4 cells that were transfected respectively with recombinant plasmids including pGL3-E, pGL3-E-142a, and pGL3-E-142a-A/B/C, and subsequently treated with *Tg*Ag for 24 h, were measured by luciferase reporter assay at 48 h-post transfection. Data were representative of the results of three independent experiments (mean ± SD). Statistical analysis was conducted using one-way ANOVA Tukey’s multiple comparisons test. *: *P* < 0.05; n.s.: *P* > 0.05

Aiming to determine the effect of *Tg*Ag on miR-142a promoter activity, full-length sequence of miR-142a promoter (-1950/+150 bp) was inserted into pGL3-Enhancer plasmids to construct recombinant plasmids, called as pGL3-E-142a (-1950/+150). Dual luciferase activity assays were carried out to evaluate the miR-142a promoter activity in EL4 cells in the presence or absence of *Tg*Ag. The analysis showed that *Tg*Ag significantly increased the activity of the miR-142a promoter by nearly 2-fold. In contrast, the alteration in luciferase activity was negligible in the pGL3-E-transfected group (Fig. 3a). To gain further insights into the regulation of miR-142a transcription, a 2100 bp (-1950/+150) of miR-142a promoter was subjected to progressive deletions. Three truncated fragments of miR-142a promoter were respectively inserted into pGL3-Enhancer plasmids to construct recombinant plasmids, called as pGL3-E-142a-A (-1752/+150 bp), pGL3-E-142a-B (-1317/+150 bp) and pGL3-E-142a-C (-858/+150 bp) in Fig. 3b.

Through the design of successive deletions in the 5’-flanking promoter region of miR-142a, it was observed that the promoter activity was low in the − 1317/+150 and − 858/+150 regions (Fig. 3c). The highest promoter activity was found in the − 1752/+150 region, suggesting that the binding element site may be located within the − 1752 to -1317 region. Furthermore, when EL4 cells were transfected with pGL3-E-142a-A plasmids, the promoter activity was significantly higher in *Tg*Ag-stimulated EL4 cells. However, no change in promoter activity was observed in the − 1317/+150 and − 858/+150 regions upon *Tg*Ag treatment (Fig. 3d). As expected, *Tg*Ag noticeably increased the promoter activity of miR-142a in EL4 cells via targeting miR-142a promoter region (-1752 to -1317), which may contain the potential transcription factors (TFs)-responsive elements to promote the promoter activity of miR-142a.

### Antigens of *T. gondii* inhibit P53 expression

**Fig. 4:**
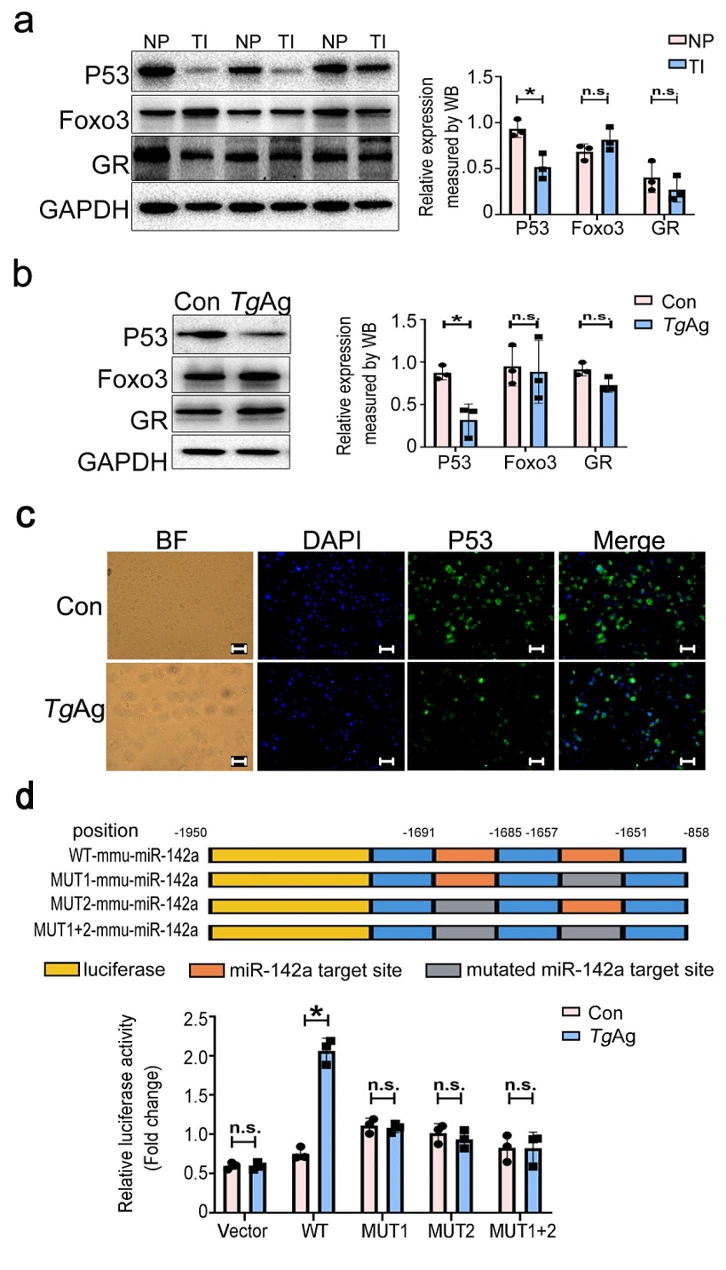
The inhibitory effects of *T. gondii* and its antigens on P53. **a** The expressions of P53, GR, and Foxo3 in mouse placentas infected with *T. gondii* were assessed by western blot (*n* = 3 mice). **b** Western blot analysis was conducted to validate the expressions of P53, GR, and Foxo3 in EL4 cells stimulated by *Tg*Ag for 24 h in vitro. **c** Immunofluorescence was conducted to visualize P53 expression in EL4 cells stimulated with *Tg*Ag for 24 h. As for fluorescence microscope image, Dye-green and Hoechst was used to label Foxp3 (green) and nuclei (blue). Scale: 40 μm. **d** The luciferase activity in EL4 cells that were transfected respectively with recombinant plasmids containing the WT or mutations (Mut 1, Mut 2, and Mut 1 + 2) sequences of miR-142a promoter was detected by luciferase reporter assay at 48 h-post transfection. *Tg*Ag: *T. gondii* antigens. NP: normal pregnant mice; TI: *Toxoplasma*-infected pregnant mice. Data were representative of the results of three independent experiments (mean ± SD). Statistical analysis was conducted using two-tailed unpaired Student’s *t*-test. *: *P* < 0.05; n.s.: *P* > 0.05

The potential TFs were predicted and analyzed to be within the active region of miR-142a promoter (-1752 to -1317) using PROMO (http://www.lsi.upc.es/~alggen) as well as Jaspar (https://jaspar.genereg.net/). We used Western blot to confirm the effect of *T. gondii* and its antigens on the involved potential TFs, including glucocorticoid receptor (GR), forkhead box O3 (Foxo3), and tumor protein P53. A significant reduction was observed in P53 protein levels in the placenta of *Toxoplasma*-infected mice, while GR and Foxo3 protein levels remained unchanged (Fig. 4a). The similar phenotype was found in *Tg*Ag-stimulated EL4 cells (Fig. 4b), suggesting the inhibitory effect of *T. gondii* and its antigens on P53. Subsequently, we detected P53 expression by IF in EL4 cells stimulated with *Tg*Ag in vitro for 24 h. These results indicated that fluorescence intensity of P53 in the *Tg*Ag-stimulated group was markedly weakened vs. that in the control group, further confirming the inhibitory effect of *Tg*Ag on P53 expression (Fig. 4c).

Afterward, we use PROMO database to analyze the underlying binding sites within the upstream of miR-142a promoter region and P53. The results unveiled that P53 had two potential binding sites (nucleotides − 1691/–1685 and − 1657/–1651) on the miR-142a 5’-flank promoter, which is consistent with the analysis of promoter activity in EL4 cells that were transfected with the plasmids containing the truncated fragments of miR-142a promoter. Then, we mutated one or both of them to determine which site was functional. *Tg*Ag administration resulted in an increase in the luciferase activity in EL4 cells that were transfected with pGL3-E-142a, while it had no effect on the luciferase activity in EL4 cells that were respectively transfected with pGL3-E-142a-A-Mut 1, pGL3-E-142a-A-Mut 2 or pGL3-E-142a-A-Mut 1 + 2 (Fig. 4d). Altogether, these data suggest that the treatment of *Tg*Ag leads to a notably increase in the promoter activity of miR-142a in a P53-dependent manner in which P53 binds to the involved regions of miR-142a promoter (–1691/–1685 and − 1657/–1651).

### miR-142a directly targeted Foxp3 3’UTR

**Fig. 5:**
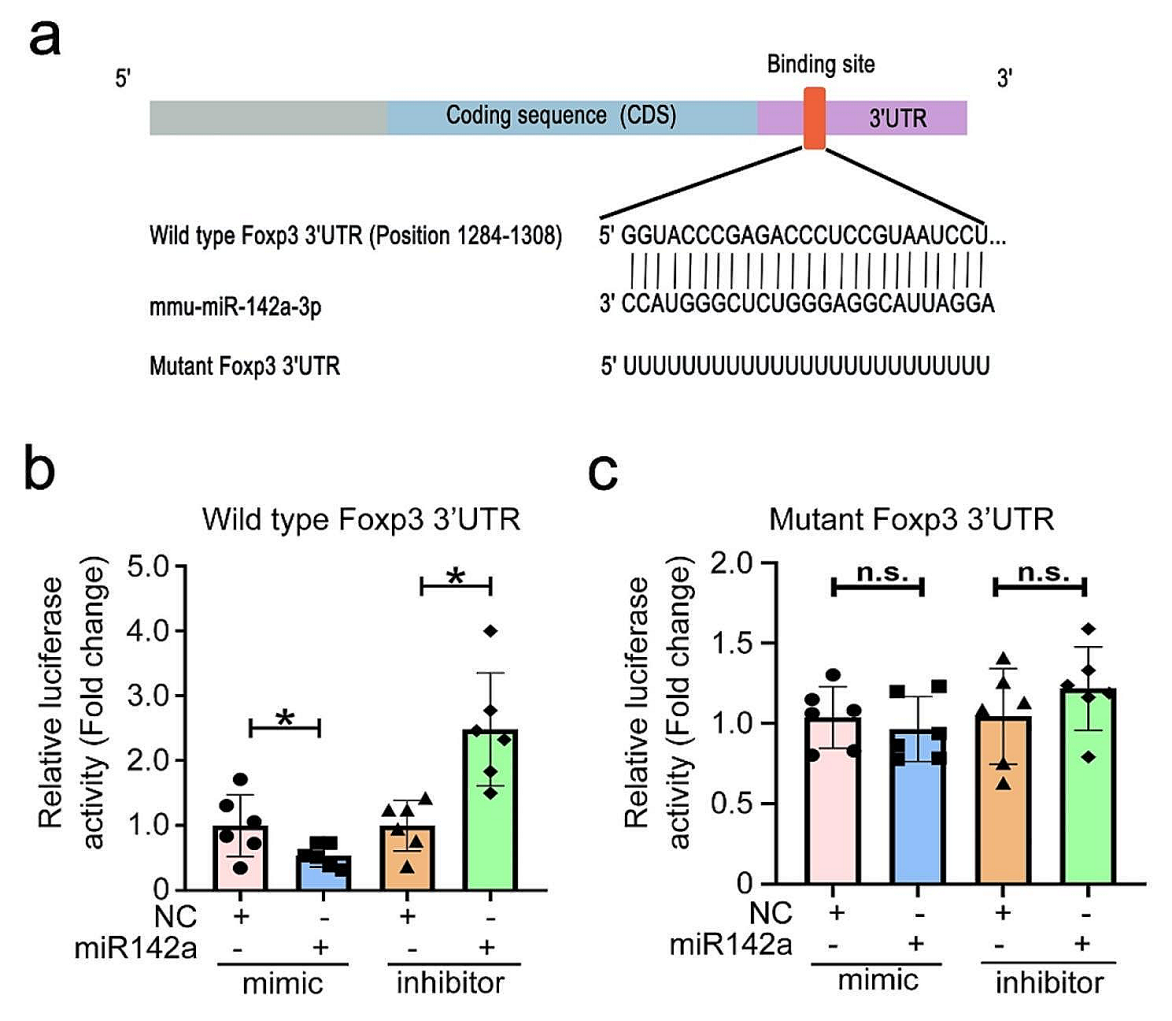
miR-142a targeted Foxp3 3’UTR. **a** Database prediction of miR-142a binding site on Foxp3 3’UTR and schematic representation of the mutation site. **b** Effect of miR-142a mimics and inhibitors on the fluorescence activity in EL4 cells transfected with recombinant plasmids containing wild type Foxp3 3’UTR. **c** Effect of miR-142a mimics and inhibitors on fluorescence activity of EL4 cells that were transfected with recombinant plasmids containing mutant Foxp3 3’UTR. Data were representative of the results of three independent experiments (mean ± SD). Statistical analysis was conducted using one-way ANOVA Tukey’s multiple comparisons test. *: *P* < 0.05; n.s.: *P* > 0.05

Having established the direct involvement of miR-142a in Foxp3 regulation, to unravel the molecular mechanism by which miR-142a suppressed Foxp3 expression, the bioinformatics miRWalk website was utilized to predict the binding site between Foxp3 3’UTR and miR-142a. As shown in Fig. 5a, Foxp3 3’UTR contains a specific region (nucleotides 1284–1308), which perfectly complements the “seed” region of miR-142a. EL4 cell was co-transfected with recombinant plasmids containing Foxp3-WT/MUT and miR-142a mimic/inhibitor, respectively. After 48 h of culture, the interaction between Foxp3 and miR-142a was verified using dual luciferase reporter assay. It showed that co-transfection of miR-142a mimic and Foxp3-WT markedly repressed luciferase activity, whereas co-transfection of miR-142a inhibitor and Foxp3-WT prominently enhanced the luciferase activity in EL4 cells (Fig. 5b). Likewise, no significant difference of luciferase activity was found in Foxp3-MUT groups with miR-142a mimic or inhibitor (Fig. 5c). In conclusion, these data suggest that miR-142a negatively governs Foxp3 expression via binding to Foxp3 3’UTR.

## Discussion

*T. gondii* infection might be responsible for miscarriages or neonatal abnormalities after primary infection during pregnancy [34, 35]. In present study, mouse model of *T. gondii*-induced adverse pregnancy was constructed via intraperitoneally injection of parasite tachyzoites. Using tissue cysts or oocysts to orally infect the mice is an appropriate way to construct the animal model, which is one of the common sources of infection in nature. Both oral and intraperitoneal infections can result in host immunity and may differ in the timing of triggering the host immune response. Nevertheless, there is no evidence to confirm any difference resulting from the two infection ways in terms of host immune response, especially maternal-fetal immune tolerance. Currently, intraperitoneal way to infect the mice is often being used for studies on the mechanism of adverse pregnancy caused by *T. gondii* [36].

miR-142a, highly conserved in vertebrates, is implicated in a number of physiological processes. miR-142-3p, highly expressed in thymic-derived regulatory T cell (tTreg), potentially targets autophagy-related protein 16 − 1 (ATG16L1) to regulate autophagy [37]. miR-142a-3p exerts as a protective role in *Escherichia coli-*induced acute lung injury via down-regulation of NF-κB signaling pathway [38]. As a key regulator in development and proliferation of mouse thymocyte, lack of miR-142 affects the expression of cell cycle-promoting genes in mature mouse thymocytes, which is accompanied by increased cyclin-dependent kinase inhibitor 1B (Cdkn1b) [39]. Yet, no studies have been reported on miR-142a functions in *T. gondii*-induced adverse pregnancy and its principal underlying mechanism. Via detecting miR-142a expression, we found that miR-142a expression was obviously increased when pregnancy mice were infected by *T. gondii* or EL4 cells were stimulated with *Tg*Ag, whereas Foxp3 expression was noticeably reduced. It promotes us to investigate the relationship between the increased miR-142a and decreased Foxp3. We uncovered that miR-142a mimic inhibited Foxp3 expression, while its inhibitor could promote Foxp3 expression in vitro.

Previous studies have provided more inclusive data on the immunomodulation of Foxp3 by miRNAs, mainly exerting inhibitory effects. miR-206 facilitates Th17 differentiation but attenuates Treg cell differentiation by targeting the 3’UTR of suppressor of cytokine signaling 3 (SOCS3) and Foxp3, respectively repressing the expressions of SOCS3 and Foxp3 [40]. miR-210 targets Foxp3 3’UTR to repress its expression and tune the immunosuppressive function of Tregs in Graves’ disease [41]. miR-142-3p tunes Foxp3 and inhibits its function by targeting autophagy-related protein 16 like protein 1 (ATG16L1) gene. miR-142-3p knockdown caused upregulation of ATG16L1 expression, resulting in higher suppressive function and enhanced autophagy of thymus-derived Treg (tTreg) cells [42]. However, it is pointed out that miRNAs can also stabilize translation complexes and promote protein production. The increased levels of miRNA-21 and miRNA-10a were observed in CD4^+^CD25^+^ T cells as compared to CD4^+^CD25^-^ T cells, indicating that these two miRNAs could enhance the inhibitory function of Treg cells by stabilizing Foxp3 [43]. In our current study, in vitro results showed that miR-142a mimic inhibited Foxp3 expression while its inhibitor could promote Foxp3 expression, suggesting that miR-142a can negatively govern Foxp3 expression. Hence, miR-142a inhibited Foxp3, but not stabilized Foxp3 in the context of *T. gondii* infection. Understanding the regulatory mechanism by which *T. gondii*-induced miR-142a suppresses Foxp3 expression can provide new perspectives for early diagnosis of *T. gondii*-induced adverse pregnancy outcomes.

P53 (also known as TP53 in humans and TrP53 in mice), a canonical tumor suppressor, acts as an oncogenic modulator and mediates intracellular repair in different cell types [44, 45]. P53 functionally tunes transcriptional regulation including miRNA genes [46]. P53 induces up-regulation of miR-194 expression in THBS1 retrovirus-induced HCT116 cells, consequently, with the decreased thrombospondin-1 (TSP-1) [47]. P53 targets binding sites in the pantothenate kinase 1 (PANK1) promoter to activate the PANK1 gene and transcriptionally stimulate miRNA-107 expression [48]. P53 directly binds to miR-128 promoter and miR-200c promoter to enhance transcription [49, 50]. In addition to acting as a transcriptional activator, P53 can be a critical repressor in miRNA expression as well. P53 can bind to the miR-27a promoter region to inhibit miR-27a expression, which in turn inhibits nuclear factor of activated T-cells 5 (NFAT5) via direct targeting NFAT5 3’UTR [51]. In the study, we ascertained that only P53 was suppressed by *Tg*Ag as compared with other potential TFs like GR and Foxo3, suggesting that P53 is the bona fide regulator of miR-142a expression. Mutations in the P53 binding sites in the miR-142a promoter reduced the promoter activity of miR-142a induced by *T. gondii* antigens. Therefore, *T. gondii* antigens influence miR-142a promoter activity through a P53-dependent mechanism.

**Fig. 6:**
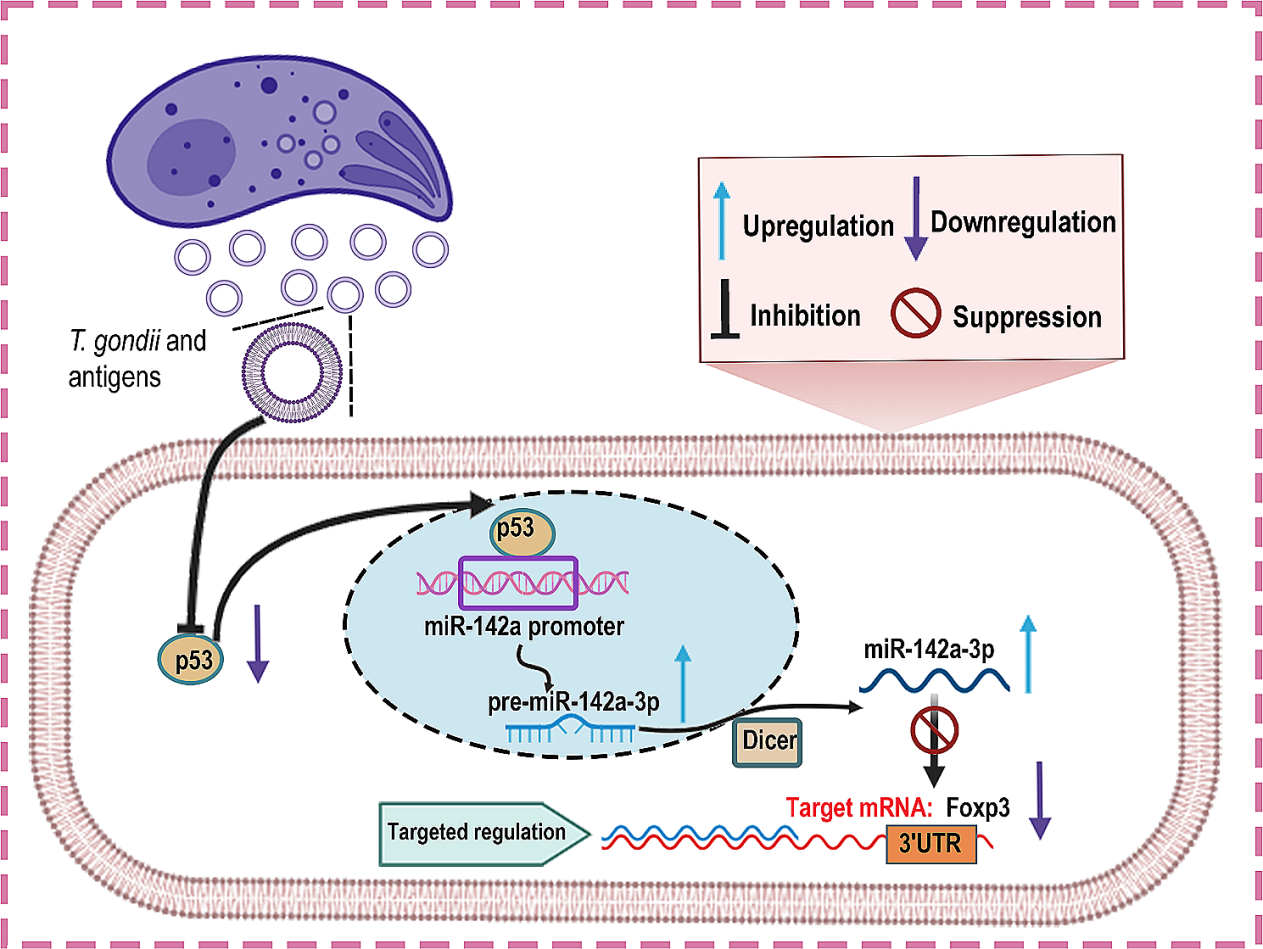
A schematic of the proposed model depicting a P53/miR-142a /Foxp3-mediated regulatory pathway in *T. gondii*-triggered adverse pregnancy outcomes in mice. During intracellular infection, *T. gondii* antigens lead to the downregulation of P53, which binds to miR-142a promoter region. miR-142a then targets Foxp3 3’UTR, therefore repressing Foxp3 expression

Based on our previous and current results, *T. gondii*-infected mice displayed an increase in miR-142a transcription in placenta issue, together with a decrease in Foxp3 expression. Consistently, comparable findings have been confirmed in *Tg*Ag-treated EL4 cells. Additionally, miR-142a mimic significantly inhibited Foxp3 expression in EL4 cells. Our study further revealed that miR-142a can directly bind to Foxp3 3’UTR, thereby inhibiting Foxp3 expression. Our data suggested that *T. gondii* might influence miR-142a promoter activity via a P53-dependent mechanism, thereby negatively regulating Foxp3 expression (Fig. 6). These findings provide new perspective for the pathogenesis as well as potential therapeutic targets for adverse pregnancy outcomes.

## Conclusion

Different sources of miRNAs inhibit Foxp3 expression during *T. gondii* infection, demonstrating the complexity of the infection process of this parasite. Our research reveals a negative correlation between up-regulation of miR-142a and down-regulation of Foxp3 in adverse pregnancy outcomes caused by *T. gondii*, potentially unveiling new molecular mechanisms involving miR-142a-mediated regulation of Foxp3 expression. With the discovery of more and more miRNA-Foxp3 interactions, investigating the role of miRNAs like miR-142a in controlling Foxp3 expression, may provide important insights into the mechanisms of immune regulation and offer valuable insights into the clinical treatment and prevention of adverse pregnancy triggered by *T. gondii*.

## Data Availability

Data is provided within the manuscript or supplementary information files.

## Abbreviations

T. gondii: Toxoplasma gondii
Treg: Regulatory T
Foxp3: Forkhead box p3
miR-142a: microRNA-142a-3p
3’ UTR: 3’ untranslated region
TgAg: T. gondii antigens
GR: Glucocorticoid
Foxo3: Forkhead box O3
PID: Primary immunodeficiency
SNPs: Single nucleotide polymorphisms
SOCS3: Suppressor of cytokine signaling 3
tTreg: Thymus-derived Treg
ATG16L1: Autophagy-related protein 16 like protein 1
PANK1: Pantothenate kinase 1
TSP-1: Thrombospondin-1
NFAT5: Nuclear factor of activated T-cells 5
